# Modified regional citrate anticoagulation is optimal for hemodialysis in patients at high risk of bleeding: a prospective randomized study of three anticoagulation strategies

**DOI:** 10.1186/s12882-019-1661-y

**Published:** 2019-12-19

**Authors:** Ting Lin, Li Song, Renwei Huang, Ying Huang, Shuifu Tang, Qizhan Lin, Ying Zhang, Xingbo Wu, Hui Liang, Yuchi Wu, Yuanhan Chen, Huaban Liang, Jianchao Ma, Zhonglin Feng, Zhuo Li, Lixia Xu, Xia Fu, Zhiming Ye, Shuangxin Liu, Xinling Liang

**Affiliations:** 1 Department of Nephrology, Guangdong Provincial People’s Hospital, Guangdong Academy of Medical Sciences, Guangzhou, China; 2 Department of Nephrology, The Second Affiliated Hospital of Guangzhou Medical University, Guangzhou, China; 3 Department of Nephrology, First Affiliated Hospital of Guangzhou University of Chinese Medicine, Guangzhou, China; 4 Department of Hemodialysis, Guangdong Provincial Hospital of Chinese Medicine, Guangzhou University of Chinese Medicine, Guangzhou, China

## Abstract

**Background:**

Recommended regular saline flushing presents clinical ineffectiveness for hemodialysis (HD) patients at high risk of bleeding with heparin contraindication. Regional citrate anticoagulation (RCA) has previously been used with a Ca^2+^ containing dialysate with prefiltered citrate in one arm (RCA-one). However, anticoagulation is not always achievable and up to 40% results in serious clotting in the venous expansion chamber. In this study, we have transferred one-quarter of the TSC from the prefiltered to the post filter based on RCA-one, which we have called RCA-two. The objective of this study was to compare the efficacy and safety of RCA-two with either saline flushing or RCA-one in HD patients with a high bleeding risk.

**Method:**

In this investigator-initiated, multicenter, controlled, prospective, randomized clinical trial, 52 HD patients (77 sessions) were randomized to the RCA-2 and RCA-one group in part one of the trial, and 45 patients (64 sessions) were randomized to the RCA-2 and saline group in part two of the trial. Serious clotting events, adverse events and blood analyses were recorded.

**Results:**

**S**erious clotting events in the RCA-two group were significantly lower compared with the RCA-one and saline group (7.89% vs. 30.77%, *P* = 0.011; 3.03% vs. 54.84%, *P* < 0.001, respectively). The median circuit survival time was 240 min (IQR 240 to 240) in the RCA-two group, was significantly longer than 230 min (IQR 155 to 240, *P* < 0.001) in the RCA-one group and 210 min (IQR 135 to 240, *P* = 0.003) in the saline group. The majority of the AEs were hypotension, hypoglycemia and chest tightness, most of which were mild in intensity. Eight patients (20.51%) in the RCA-one group, 4 patients (12.90%) in the saline group and 10 patients (26.31%) in the RCA-two group, *P* > 0.05.

**Conclusions:**

Our data demonstrated that the modified anticoagulation protocol was more effective and feasible during hemodialysis therapy for patients at high risk of bleeding.

**Trial registration:**

GDREC, GDREC2017250H. Registered February 2, 2018; retrospectively registered.

## Background

**Fig. 1:**
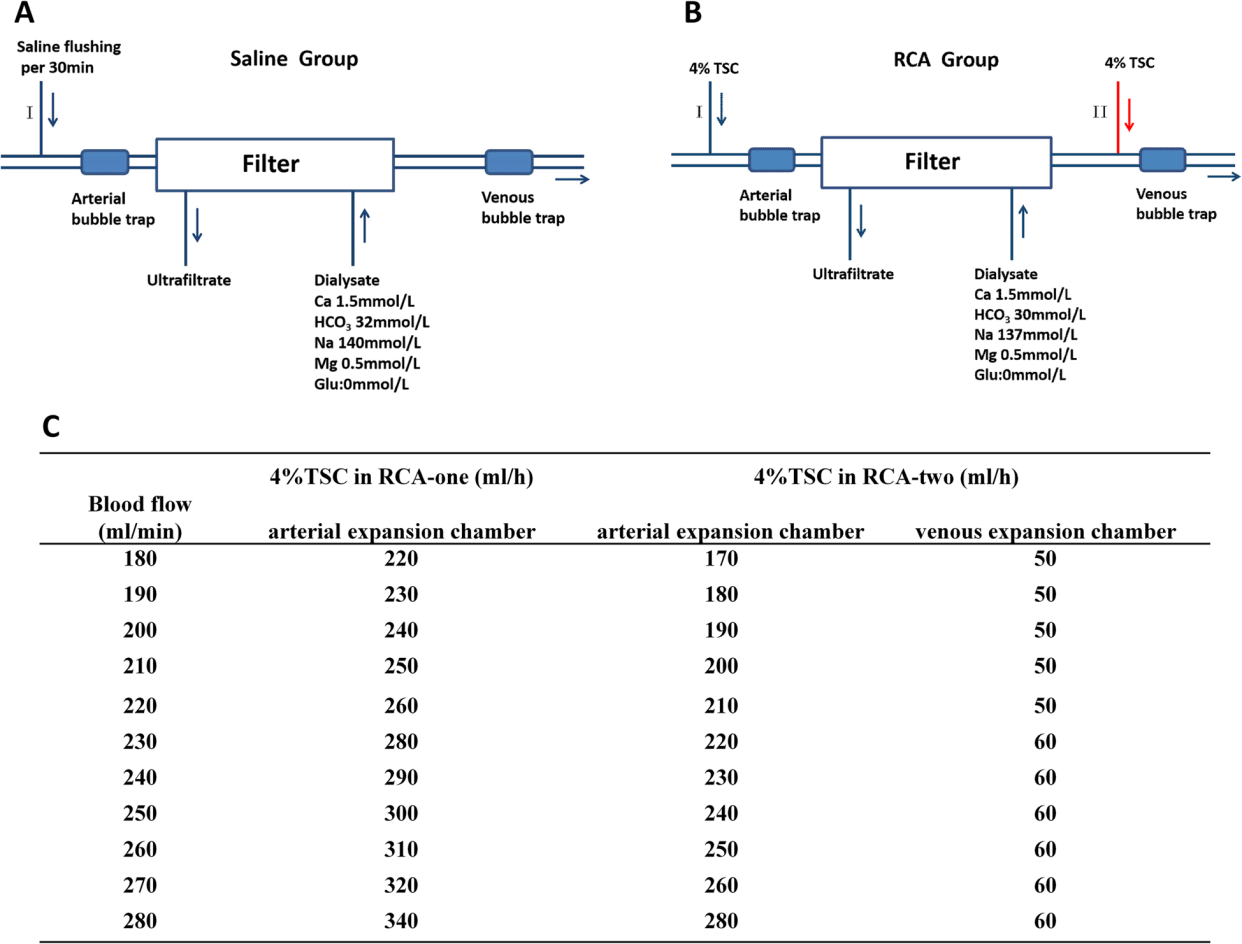
Schematized extracorporeal circuit and dialysate parameters for three anticoagulation strategies. **a** the Saline group. **b** RCA groups. RCA-one: 4% TSC was only continuously infused in position I; RCA-two: 4% TSC was infused in position I and II, simultaneously. **c** The infusion rate of 4% TSC in RCA-one and RCA-two groups. Abbreviations: RCA, regional citrate anticoagulation; TSC, trisodium citrate.

Chronic kidney disease (CKD) affects 10.8% of the population, and over 460,000 patients with CKD eventually suffer from chronic hemodialysis (HD) in China [1]. Shorter length dialysis sessions are associated with increased mortality in HD patients [2]. Accurate anticoagulation minimizes blood clotting in the extracorporeal circuit (ECC) and is essential for the efficacy of HD. According to the Kidney Disease Improving Global Outcomes 2012 (KDIGO 2012) guidelines, heparin is recommended in intermittent HD procedures, but not for patients at high risk of bleeding for whom regular saline flushing (Saline, Fig. 1a) of ECC is recommended [3]. However, the clotting event rate with this approach exceeds up to 50%, leading to frequent circuit replacement and overconsumption of platelets or erythrocytes [4]. Thus, a therapeutic strategy for prolonging the ECC lifetime in patients at high risk of bleeding is desirable both economically and for effective dialysis.

Regional citrate anticoagulation (RCA) was introduced into the hemodialysis procedure in 1983 for its excellent anticoagulation properties and reduction in bleeding complications [5]. During hemodialysis trisodium citrate (TSC) is introduced at the beginning of the ECC and it binds to calcium (Ca^2+^) and magnesium (Mg^2+^) ions, the resulting absence of Ca^2+^ in the blood causes the anticoagulation effect. Usually an adequate amount of Ca^2+^ should be supplemented back to the systemic circulation according to the amount of imported TSC. Since 2012, RCA has been recommended as a priority anticoagulant for patients who require continuous renal replacement therapy (CRRT) because studies have shown that RCA has better anticoagulation properties and less bleeding risk than heparin [6–8].

In recent years, several studies have shown the effectiveness of RCA during intermittent HD procedures. RCA using Ca-free dialysate revealed no serious clotting events; however, electrolytes need to be tested frequently and Ca^2+^ supplementation is required, eventually limiting its clinical application [9–11]. However, when RCA with a Ca^2+^ containing dialysate with prefiltered citrate in one arm (RCA-one) is used, Ca^2+^ supplementation is unnecessary owing to restoration of Ca^2+^ by the Ca^2+^-containing dialysate. Though, anticoagulation is not always achievable and up to 40% results in serious clotting in the venous expansion chamber [9–11]. It is critical to further explore a superior anticoagulation approach to achieve both effectiveness and feasibility.

We have previously tested the concentration of iCa^2+^ at several points of the ECC in RCA-one (Additional file 5: Figure S5) and found that the concentration of iCa^2+^ is particularly high in the venous bubble trap, which would be the key point of clotting and found that the concentration of iCa^2+^ is particularly high in the venous bubble trap, which would be the key point of clotting. Given this consideration, we have transferred one-quarter of the TSC from the prefiltered to the post filter based on RCA-one, which is called RCA-two in this trial (Fig. 1b).

The objective of this study was to compare the efficacy and safety of RCA-two with either saline flushing or RCA-one in HD patients with a high bleeding risk.

## Methods

### Design

This investigator-initiated, multicenter, placebo-controlled, prospective, randomized clinical trial evaluated the efficacy and safety of RCA-two in a population of intermittent HD patients with a high risk of hemorrhage. Ethical approval of the study protocol was obtained from the institutional review boards of each participating hospital, including Guangdong Provincial People’s Hospital (Project Number: 2017250H (R1)), the First Affiliated Hospital of Guangzhou University of Chinese Medicine (Project Number: ZYYECK [2018]008), the Second Affiliated Hospital of Guangzhou Medicine University (Project Number: 2018-hs-06), and Guangzhou Hospital of Chinese Medicine (Project Number: B2017–189-01). The trial was prospectively registered at the international clinical trial registry system with the identifier GDREC2017250H (https://www.clinicaltrials.gov/ct2/show/NCT03419923?term=GDREC2017250H&rank=1).

### Inclusion and excluded criteria

Eligible patients were 18 years old or older and suffered from CKD requiring HD with a high risk of bleeding. The HD procedure should carried out with a dialysis fluid more than 500 ml/min and blood flow volume more than 3.5-4 ml/min.kg. The definition of high risk of bleeding was defined according to the criteria in previous reports [12] as active hemorrhage (within 3 days), pre-invasive operation (within 7 days), and post-operation (within 3 days). Patients were excluded if they had a high risk of citrate accumulation, defined as total bilirubin greater than 60 μmol/L, lactic acid > 3 mmol/L; a usage of the drugs that impact the coagulation function within 7 days; and severe hypocalcemia defined as a serum calcium concentration less than 1.9 mmol/L. Written informed consent was obtained from all subjects prior to enrollment and participation. This trial was complied with the Declaration of Helsink and adhered to the International Conference on Harmonisation Guidelines on Good Clinical Practice.

#### Randomization and interventions

**Fig. 2:**
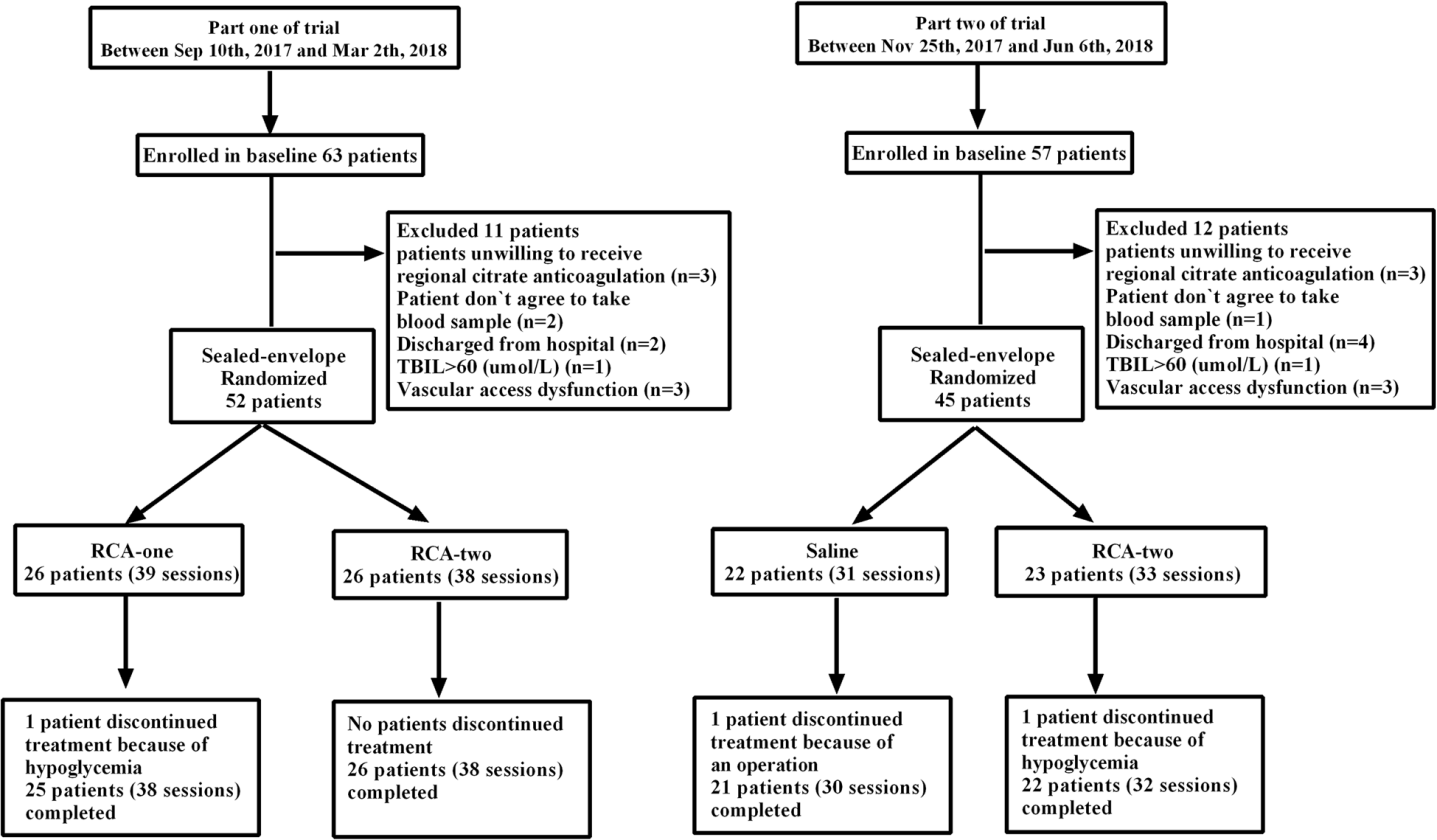
Patient flow of the trial. Abbreviations: Saline, saline flushing group; RCA-two, two-stage regional citrate anticoagulation group; RCA-one, one-stage regional citrate anticoagulation group; n: number of patients.

There were two parts to this trial. In part one, patients were randomized to the RCA-one or RCA-two group in a 1:1 ratio; in part two, patients were consecutively randomized to the Saline or RCA-two group in a 1:1 ratio **(Fig.** 2**).** *RCA-one group:* A sterile solution of trisodium citrate (4% TSC; 136 mmol/L; ChengDu QiShan LiKang Pharmaceutical CO. LTD., SiChuan, China) was continuously infused at the beginning of the arterial line. *RCA-two group:* 4% TSC was infused in two stages: at the beginning of the arterial line and at the venous expansion chamber (Fig. 1**b**). Generally, three-quarters of the total 4% TSC was infused at the beginning of the arterial line, and one-quarter was infused at the venous expansion chamber. The rate of 4% TSC was calculated according to the blood flow supplemented in Fig. 1**c**. *Saline group:* 0.9% saline flushes with 250 mL were carried out through the arterial line every 30 min (Fig. 1**a**).

The process of randomization was using concealed opaque envelopes. The randomization sequence was computer generated by a statistician. Sequence were enclosed in opaque sealed envelopes. After written informed consent was obtained, one envelope was randomly drawn and opened by the investigator. Patients were withdrawn from the study protocol when bleeding risk had disappeared or after completion of 3 sessions of dialysis treatment or discharged. Patients, treating physicians and nurses were not blinded to the patients’ treatment and Clotting of each circuit. Apart from the trial statistician and the data-monitoring committee, all treating physicians and nurses remained blinded to the trial results until this trial was completed.

#### Hemodialysis parameters

High-flux HD therapy for 4 h with a dialysate flow of 500 mL/min was delivered using a center dialysate delivery system (4008S; Fresenius Medical Care, Germany). The dialyzers applied in this study were triacetate (TAT) membrane (hollow-fiber, FB-170 U, 1.7 m^2^, Nipro, Japan) or polyethersulfone (PES) membrane (hollow-fiber, ELSIO.17H, 1.7 m^2^, Nipro, Japan), which were reported to have similar thrombogenicity and sieving coefficients [13]. The detail dialysate parameters were shown in Fig. 1a and b. The blood flow was maintained at approximately 180–250 ml/min. Circuits and dialyzers were primed using 1.0 L of 0.9% saline without a heparin pre-dialysis procedure. At the end of the hemodialysis, 4% TSC was used for locking the catheter’s device.

#### Data collection

Demographics and baseline characteristics were collected from electronic databases in the purification centers of each participating hospital. Adverse events (AEs) were assessed by physicians throughout the study. Blood analyses were performed at 0, 1, 2 and 4 h during the HD session. Arterial pressure, venous pressure and transmembrane pressure (TMP) were observed routinely by nurses throughout HD therapy and measured per hour. At the end of each HD procedure, the clotting scores of the arterial, venous expansion chamber and the dialyzer were evaluated through a semi-quantitative method with a range of 0 to 3 (Additional file 6: Table S1) by two experienced nurses and one physician in a blinded method. Interobserver agreement with respect to the total clotting score of the extracorporeal circuit was very high (*R*^*2*^ = 0.971, *P* < 0.001) in part one and (*R*^*2*^ = 0.976, *P* < 0.001) in part two.

### Outcomes definition

The primary outcome was measured as therapy interruption based on visible serious circuit clotting or persistent alarms such as venous pressure (> 200 mmHg) or TMP (> 300 mmHg). The secondary outcomes included circuit survival time (the lifetime of the circuit survival was limited to 240 min, because achieved the goals of treatment), the total clotting score of ECC and urea clearance (Kt/V and URR). The total clotting score was the sum of the clotting scores for the venous expansion chamber, arterial expansion chamber and dialyzer (Additional file 6: Table S1). The formula used for whole body urea clearance (Kt/V) was Kt/V = −In (C_post_/C_pre_ - 0.008 * t) + (4–3.5 * C_post_/C_pre_) * UF/W, where C_post_ is the post-dialysis blood urea (mmol/liter), C_pre_ is the pre-dialysis blood urea (mmol/liter), t is the dialysis session length (hours), UF is the total volume of ultrafiltrate (liters), and W is the post-dialysis weight (kg). The formula used for urea excretion ratio (URR) was URR (%) = (C_pre_ -C_post_)/C_pre_*100, where C_post_ is the post-dialysis blood urea (mmol/liter) and C_pre_ is the pre-dialysis blood urea (mmol/liter).

### Sample size

Based on previous trials, the clinical risk of circuit clotting with RCA-one was approximately 36% during dialysis treatment compared with 45–50% with saline flushes [14, 15]. Our clinical preliminary experiment showed that the incidence of circuit clotting with RCA-two was 10%. According to the above data, we aimed to include 48 dialysis sessions in part one and 78 sessions in part two at 80% power and an error of 5%, using the PASS software (NCSS, version 11.0.7, LLC.).

### Statistical analysis

Statistical analyses followed the protocol and the primary analysis was intention-to-treat and involved all patients who were randomly assigned. Categorical variables were described as frequencies (n) or percentages (%) and analyzed with Pearson’s chi-square or Fisher’s exact test. The Kolmogorov-Smirnov test was used to check the normal distribution of all continuous data. Parametric continuous parameters are expressed as mean ± standard deviation and analyzed with unpaired Student’s t-tests; nonparametric continuous parameters are expressed as medians (interquartile range, IQR) and analyzed with the Wilcoxon test.

A Kaplan-Meier survival curve was drawn to compare the circuit survival time using the log-rank test. The sensitivity analyses for the primary outcome (effect of RCA-two on the time to clotting) were performed used Cox proportional hazards models to adjust potential confounding variables. The main purpose of this analysis was to address potential differential competing risks from other causes of clotting between groups. To reduce the possibility that the same associated variable entered the multivariate model simultaneously, the correlation analysis between the factors screened using univariate analysis was performed. If the Spearman’s correlation coefficient between variables exceeds 0.60, only the variables considering being more important on a clinical basis were entered into the multivariate model. Variables with similar functions were chosen using the likelihood ratio test, which favored those variables with higher statistical values. If there were nonlinear association between continuous variables and the circuit survival, then the continuous variables were divided into clinically appropriate categories based on the lower quartile (Q_L_) and upper quartile (Q_U_). The main purpose of this analysis was to avoid the chances that the selected continuous variables violate linearity assumption. The Kaplan-Meier survival curves were drawn for each potential variables and the proportional hazards assumption on the basis of Schoenfeld residuals was tested. We accepted departure from the proportional hazards assumption as long as log-rank curves did not cross for the first 4 h.

The mixed models for repeated measures with an unstructured residual covariance matrix were used to analyze the change of concentrations of serum-ionized calcium from baseline to endpoint by restricted maximum likelihood [16]. The model included treatment of anticoagulation (group effect) and time (repeated measure for time effect) as fixed factors and baseline serum-ionized calcium as covariate, with interactions (interaction effect) between treatment and time included. Adjusted *P*-values for multiple Comparisons using modified Bonferroni method to analyze the differences between anticoagulation groups at each time points by unpaired Student’s t test.

Statistical significance was assumed if the 2-sided P-value was less than 0.05. The SAS software program was used for statistical analysis and data cleaning (SAS Institute, version 9.3, Cary, NC). The survival curves and other graphs were made using GraphPad Prism (GraphPad Software, version 6.1.0 for Windows, San Diego, CA, USA).

## Results

### Patients and baseline characteristics

Between Sep 10, 2017, and June 6, 2018, a total of 120 patients were enrolled in the trial, and 23 patients were ineligible for the following reasons: six were unwilling to receive regional citrate anticoagulation, six were discharged from the hospital, three did not agree to give a blood sample, and eight patients met exclusion criteria. The remaining 97 patients were randomized into the trial. In part one of the experiment, 77 HD procedures in 52 patients were randomly assigned to the RCA-one or RCA-two group from Sep 10, 2017 to Mar 2, 2018; in part two of the experiment, 64 HD procedures in 45 patients were randomly assigned to the saline or RCA-two group from Nov 25, 2017 to Jun 6, 2018. Three patients unexpectedly terminated therapy for adverse events (AEs) of hypoglycemia and medical treatment (Fig. 2).

Baseline characteristics and baseline laboratory analyses were generally similar among the groups (Table 1; Additional file 7: Table S2). Compared with the RCA-two group, there were higher amounts of ultrafiltration in the saline group (*P* = 0.006) and more patients with catheters in the RCA-one group (*P* = 0.011). In the internal environment evaluation, the hemoglobin and pH value were not balanced among groups (*P* < 0.05) (Table 1).

**Table 1:** Baseline characteristics

Factors	Part one			Part two		
	RCA-one (*n* = 39)	RCA-two (*n* = 38)	*P* value	Saline (*n* = 31)	RCA-two (*n* = 33)	*P* value
Male, n (%)	18 (46.15)	19 (50.00)	0.903	18 (58.06)	16 (48.48)	0.443
Age, median (IQR), years	55 (50–63)	56 (50–64)	0.988	56 (41–70)	52 (37–65)	0.459
Weight, median (IQR), kg	59.5 (47.0–63.0)	53.7 (50.8–60.0)	0.639	59.5 (52.0–63.3)	56.4 (50.0–60.0)	0.204
Diabetes mellitus, n (%)	5 (12.82)	6 (15.79)	0.709	4 (12.90)	8 (24.24)	0.245
Access type-AV fistula, n/catheter, n	20/19	30/8	0.011	19/12	23/10	0.479
Causes of high risk of bleeding			0.389			0.113
Perioperation, n (%)	23 (58.97)	26 (68.42)		15 (48.39)	10 (30.30)	
Hemorrhage, n (%)	16 (41.03)	12 (31.58)		12 (38.71)	19 (57.58)	
Blood flow rate, median (IQR), ml/min	200 (200–210)	210 (200–220)	0.089	200 (200–220)	200 (200–220)	0.946
Ultrafiltration, mean ± sd, L	1.8 ± 0.9	2.1 ± 0.9	0.334	1.97 ± 0.79	1.90 ± 0.75	0.728
Rate of Ultrafiltration, mean ± sd, L/h	0.7 ± 0.2	0.7 ± 0.2	0.294	0.9 ± 0.3	0.7 ± 0.2	0.006
Pre-dialysis SBP, mean ± sd, mmHg	155.41 ± 25.34	149.56 ± 24.09	0.348	150.52 ± 22.98	152.27 ± 24.41	0.768
Pre-dialysis DBP, mean ± sd, mmHg	79.78 ± 11.09	79.25 ± 13.08	0.862	79.52 ± 15.13	83.58 ± 18.12	0.336
Hemoglobin, g/L	87.0 (80.0–92.50)	95.5 (83.0–110.0)	0.023	97.0 (93.0–109.0)	82. 0 (78.0–94.0)	0.007
Blood platelets, 10^9^/L	195.5 (147.0–262.5)	194.5 (130.5–258.5)	0.847	173.0 (157.0–204.0)	216.0 (200.0–233.0)	0.082
Prothrombin time, second	13.7 (13.3–14.5)	13.8 (13.3–14.7)	0.893	13.6 (13.1–14.4)	13.3 (12.4–14.2)	0.435
Activated partial thromboplastin time, second	41.0 (36.8–43.2)	38.6 (36.5–40.3)	0.106	39.7 (36.8–41.5)	40.2 (37.0–43.2)	0.295
pH value	7.37 (7.34–7.39)	7.41 (7.38–7.42)	0.001	7.35 (7.33–7.39)	7.40 (7.36–7.42)	0.001

*Abbreviations*: *VP* venous pressures, *TMP* transmembrane pressures, *AP* arterial pressures, *DBP* diastolic blood pressure, *SBP* Systolic blood pressure, *AV fistula* arterial- venous fistula, *IQR* interquartile range, *n* patient, *sd* Standard deviation. Categorical variables were described as frequencies (n) or percentages (%) and analyzed with Pearson’s chi-square or Fisher’s exact test. The Kolmogorov-Smirnov test was used to check the normal distribution of all continuous data. Parametric continuous parameters are expressed as mean ± standard deviation and analyzed with unpaired Student’s t-tests; nonparametric continuous parameters are expressed as medians (interquartile range, IQR) and analyzed with the Wilcoxon test

### Primary outcomes

**Fig. 3:**
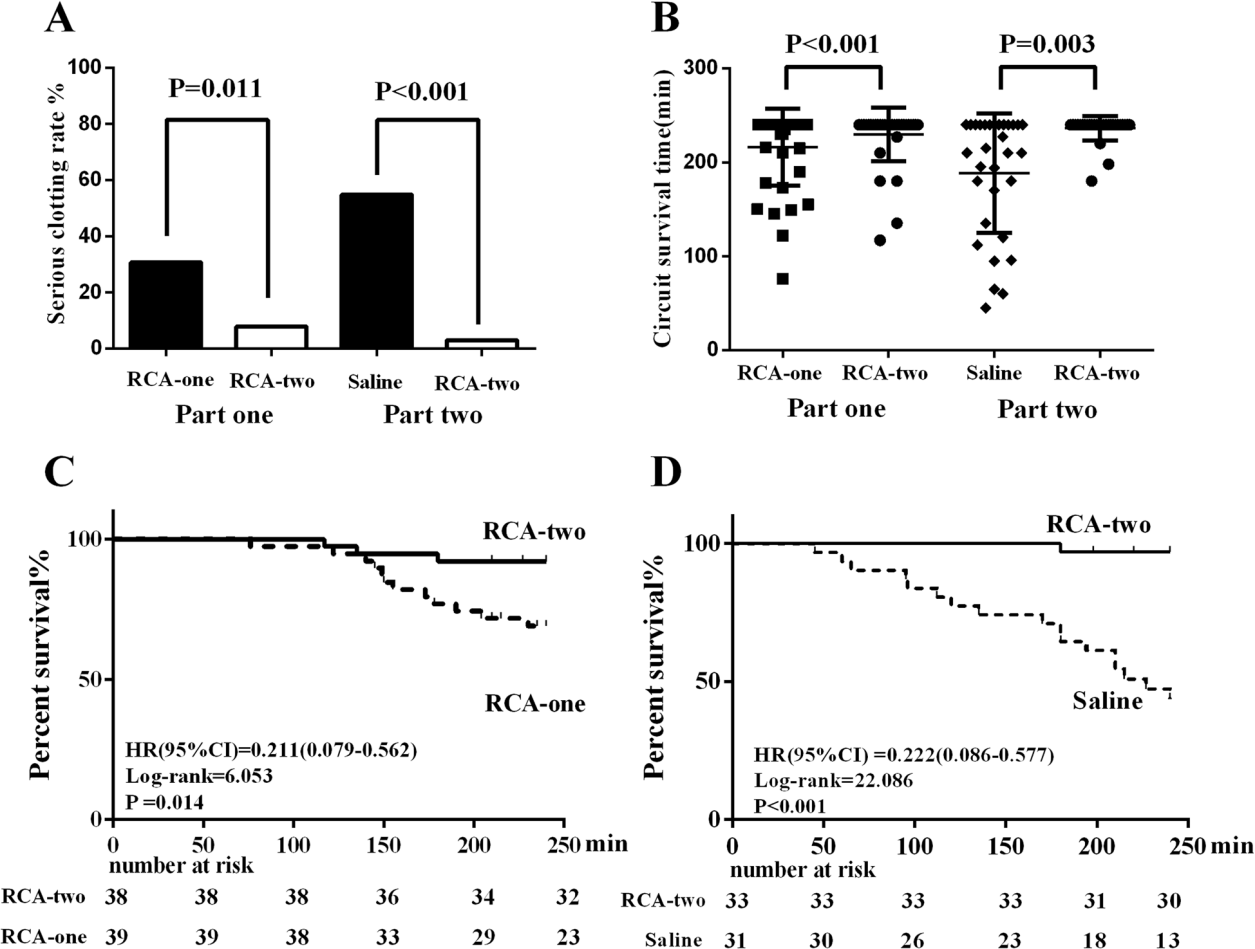
Efficacy endpoints and cumulative probability of survival for the circuit. **a** Serious clotting rate (the primary outcome) in part one and part two trial. **b** The circuit survival time in each group. The median circuit survival time was 230 min (IQR 155 to 240) in the RCA-one group vs. 240 min (IQR 240 to 240) in the RCA-two group (*P* < 0.001); The median circuit survival time was 210 min (IQR 135 to 240) in the saline group vs. 240 min (IQR 240 to 240) in the RCA-two group (*P* = 0.003). **c** The Kaplan–Meier curves for uncensored circuit survival between the RCA-two and RCA-one groups. **d** The Kaplan–Meier curves for uncensored circuit survival between the RCA-two and Saline groups. The survival analyses were based on the log-rank test. Abbreviations: Saline, saline flushing group; RCA-two, two-stage regional citrate anticoagulation group; RCA-one, one-stage regional citrate anticoagulation group

Therapy-interrupted events based on serious clotting in the RCA-two group were significantly lower than in the RCA-one and saline groups (3/38 (7.89%) vs. 12/39 (30.77%), *P* = 0.011; 1/33 (3.03%) vs. 17/31 (54.84%), *P* < 0.001, respectively) (Fig. 3a). The percentage of sessions with persistent venous pressure (> 200 mmHg), TMP (> 300 mmHg) and visible serious circuit clotting in each group were presented in Additional file 1: Figure S1A and B. The median circuit survival time was 240 min (IQR 240 to 240) in the RCA-two group, which was significantly longer than 230 min (IQR 155 to 240, *P* < 0.001) in the RCA-one group and 210 min (IQR 135 to 240, *P* = 0.003) in the saline group (Fig. 3b).

### Secondary outcomes

#### Circuit survival time

Kaplan-Meier analysis showed that the circuit survival time of the RCA-two group was higher than the RCA-one group (HR (95% CI) = 0.211 (0.079–0.562), *P* = 0.014) (Fig. 3c) and saline group (HR (95% CI) = 0.222 (0.086–0.577), *P* < 0.001) (Fig. 3d). According to the univariate analysis (Additional file 8: Table S3) and clinical observations, we included seven potential variables that might affect extracorporeal circuit survival time: the anticoagulation protocol, diabetes mellitus, blood flow, access type, pre-dialysis glucose, blood platelets, activated partial thromboplastin time, and hemoglobin.

#### Cox proportional hazard analysis

Spearman’s correlation analysis demonstrated the correlation coefficients between variables were all under 0.6 in two part trials (Additional file 2: Figure S2 and Additional file 3: Figure S3). A multivariable Cox proportional hazard analysis showed that the effect of RCA-two on circuit survival was significant, compared with RCA-one (adjusted hazard ratio (95% confidence interval) (adjusted HR (95% CI)) = 0.224 (0.084–0.598), *P* = 0.003, Table 2) and the saline (adjusted HR (95% CI) = 0.184 (0.069–0.491), *P* < 0.001, Table 2). In addition, blood flow and diabetes mellitus could also influence the anticoagulation effects (Table 2).

**Table 2:** Influencing factors of time to clot in Cox proportional-hazards models

Factors	Part one		Part two	
	Adjust (Selection = STEPWISE Likelihood Ratio = 27.333 *P* < 0.001)		Adjust (Selection = STEPWISE Likelihood Ratio = 9.327 *P* = 0.002)	
	Adjusted HR (95%CI)	*P* value	Adjusted HR (95%CI)	*P* value
RCA-two ^a^	0.224 (0.084–0.598)	0.003	0.184 (0.069–0.491)	< 0.001
Diabetes mellitus (reference Non-Diabetes mellitus)	1.675 (0.471–5.954)	0.425	2.730 (1.075–6.934)	0.035
AV fistula (reference Catheter)	0.511(0.217–1.207)	0.126	0.516 (0.187–1.420)	0.200
Blood flow < 220 ml/min (reference ≥220 ml/min)	4.297 (1.009–18.296)	0.049	1.181 (0.235–5.929)	0.840
APTT ≥45 s	0.482 (0.048–4.802)	0.534	1.834 (0.245–13.754)	0.555
35 ≤ APTT < 45 s	0.347 (0.113–1.064)	0.064	1.335 (0.359–4.972)	0.666
APTT < 35 s	reference		reference	
Blood platelets > 200 × 10^9^/L	1.758 (0.655–4.723)	0.263	1.072 (0.311–3.696)	0.912
160 ≤ Blood platelets < 200 × 10^9^/L	4.676 (0.894–24.453)	0.068	0.774 (0.159–3.775)	0.752
Blood platelets < 160 × 10^9^/L	reference		reference	
Glucose ≥8.4 mmol/L	1.744 (0.403–7.544)	0.457	0.757 (0.086–6.678)	0.803
4.4 ≤ Glucose < 8.4 mmol/L	1.832 (0.495–6.778)	0.364	1.516 (0.379–6.071)	0.557
Glucose < 4.4 mmol/L	reference		reference	
Hemoglobin ≥110 g/L	3.354 (0.447–25.170)	0.239	1.458 (0.348–6.111)	0.606
80 ≤ Hemoglobin < 110 g/L	1.054 (0.332–3.343)	0.929	0.612 (0.147–2.554)	0.501
Hemoglobin < 80 g/L	reference		reference	

The continuous variables were divided into clinically appropriate categories based on the lower quartile (QL) and upper quartile (QU). Blood flow was divided into clinically appropriate categories based on median value (220 ml/min). The main purpose of this analysis was to avoid the chances that the selected continuous variables violate linearity assumption. *Abbreviations*: *APTT* activated partial thromboplastin time, *CI* confidence interval, *AV fistula* arterial- venous fistula, *s* second

^a^RCA-one as reference in part one and Saline group as reference in part two

#### Total clotting scores and urea clearance

We also estimated the degree of clotting by calculating the clotting score at the end of dialysis. The serious clotting score (= 3) in any position (dialyzer, venous expansion chamber or arterial expansion chamber) in the RCA-two group was significantly lower than the RCA-one group and saline group (5/38 vs. 20/39, *P* < 0.001; 6/33 vs. 15/31, *P* = 0.016, respectively) (Table 3). Specifically, the differences in clotting scores for the arterial, venous expansion chamber and dialyzer were all significant between the RCA-two and saline groups (*P* < 0.001), while the only critical clotting position was the venous expansion chamber in the RCA-one group, compared with the RCA-two group (*P* < 0.001) (Additional file 1: Figure S1C and D). In addition, the estimation of KT/V and URR were similar between the RCA-two and RCA-one groups (*P* > 0.05); in part two of the experiment, KT/V and URR showed that patients in the RCA-two group had better hemodialysis efficacy than in the saline group [1.41 (1.27–1.48) vs. 1.13 (0.79–1.41), *P* = 0.007; 0.70 (0.66–0.71) vs. 0.62 (0.51–0.70), *P* = 0.005] (Table 3).

**Table 3:** Secondary outcomes in two parts of the trial

Secondary and Safety Outcomes	Part one				Part two		
	RCA-one (*n* = 39)	RCA-two (*n* = 38)	*P* value		Saline (*n* = 31)	RCA-two (*n* = 33)	*P* value
Serious clotting scores (=3) (%)	20 (51.28)	5 (13.16)	< 0.001		15 (48.39)	6 (18.18)	0.016
Arterial expansion chamber (%)	0 (0.00)	0 (0.00)	1.000		1 (3.23)	0 (0.00)	0.484
Dialyzer (%)	2 (5.13)	1 (2.63)	0.289		1 (3.23)	1 (3.03)	1.000
Venous expansion chamber (%)	18 (46.15)	4 (10.53)	< 0.001		13 (41.94)	6 (18.18)	0.038
Urea clearance							
KT/V value, median (IQR) (%)	1.25 (1.01–1.65)	1.24 (1.11–1.42)	0.792		1.13 (0.79–1.41)	1.41 (1.27–1.48)	0.007
URR value, median (IQR), %	0.65 (0.62–0.75)	0.66 (0.61–0.71)	0.488		0.62 (0.51–0.70)	0.70 (0.66–0.71)	0.005
AEs	8 (20.51)	10 (26.31)	0.547		4 (12.90)	7 (21.21)	0.379
Dizzy, n (%)	0 (0.00)	1 (2.63)	0.494		0 (0.00)	1 (3.03)	1.000
Convulsions, n (%)	0 (0.00)	1 (2.63)	0.494		1 (3.23)	1 (3.03)	1.000
Hypoglycemia, n (%)	1 (2.56)	0 (0.00)	1.000		2 (6.45)	1 (3.03)	0.607
Hypotension, n (%)	4 (10.26)	6 (15.79)	0.517		1 (3.23)	2 (6.06)	1.000
Chest tightness, n (%)	3 (7.69)	1 (2.63)	0.615		0 (0.00)	0 (0.00)	1.000
Other, n (%)	0 (0.00)	1 (2.63)	0.494		0 (0.00)	2 (6.06)	0.493

*Abbreviations*: *KT/V* whole body urea clearance, *URR* urea clearance ratio, *AEs* Adverse events, *IQR* interquartile range, *n* patient. Serious clotting scores (=3) wsa defined as Volume of thrombus more than 2/3 of expansion chamber or Area of streaky Hemofilter more than 2/3 of total. Categorical variables were described as frequencies (n) or percentages (%) and analyzed with Pearson’s chi-square or Fisher’s exact test. The Kolmogorov-Smirnov test was used to check the normal distribution of all continuous data. Parametric continuous parameters are expressed as mean ± standard deviation and analyzed with unpaired Student’s t-tests; nonparametric continuous parameters are expressed as medians (interquartile range, IQR) and analyzed with the Wilcoxon test

### Safety outcomes

#### AEs occurrence

AEs are summarized in Table 3. During the course of part one of this study, eight patients (20.51%) receiving RCA-one anticoagulation and 10 patients (26.31%) receiving RCA-two anticoagulation had complications, with no statistical differences (*P* = 0.547). The majority of the AEs were hypotension and chest tightness, most of which were mild in intensity. In part two, seven patients (21.21%) in the RCA-two group and four patients (12.90%) in the saline group had complications (*P* = 0.379). A majority of the AEs were hypoglycemia and hypotension, most of which were mild to moderate in intensity.

#### Trisodium citrate (TSC) accumulation or internal environment disturbances

**Fig. 4:**
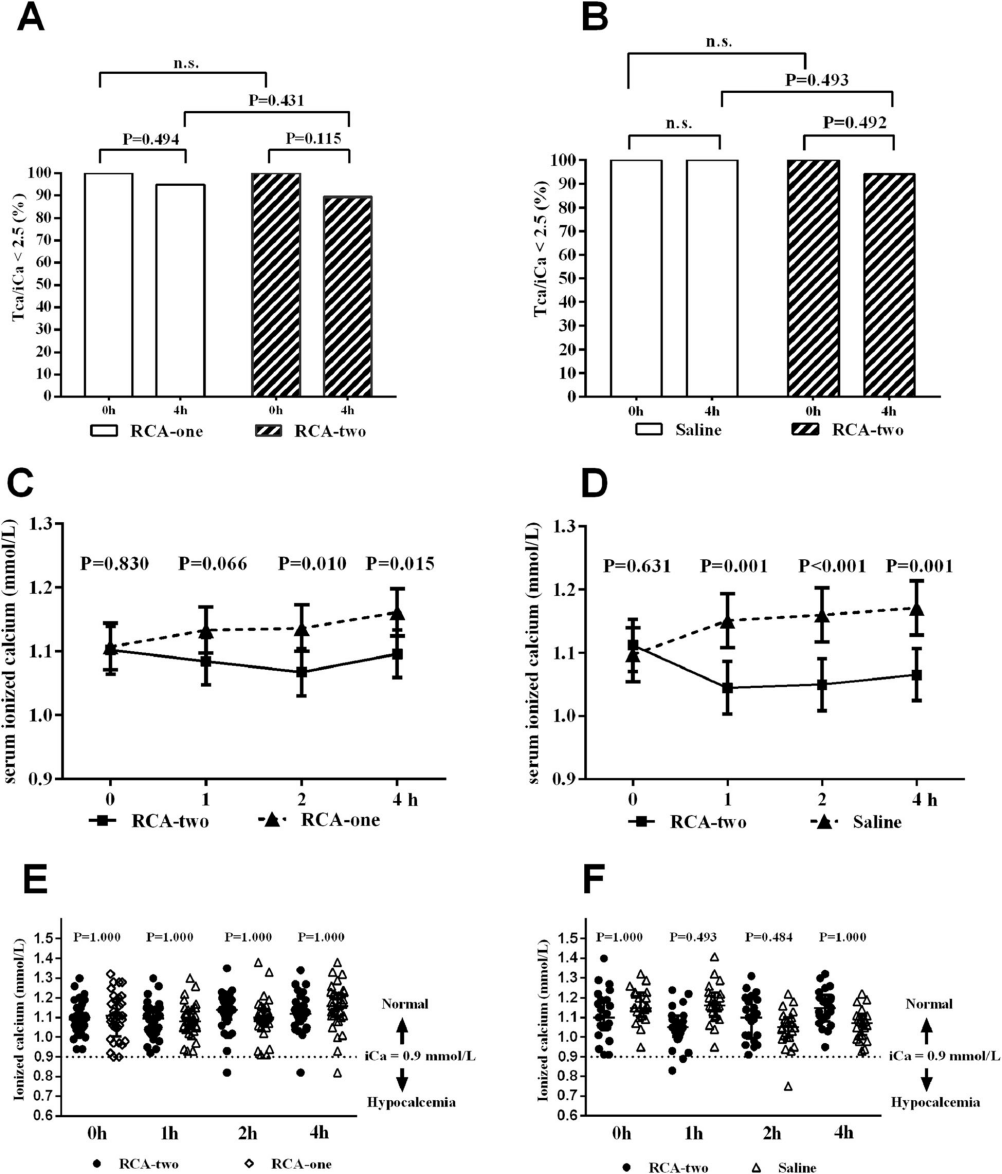
The evaluation of TSC overdose and hypocalcemia throughout the treatment. **a** The percentage of Total calcium to ionized calcium (T/I Ca^2+^) levels below 2.5 at hour 0 and 4 in the RCA-one and RCA-two groups. **b** The percentage of Total calcium to ionized calcium (T/I Ca^2+^) levels below 2.5 at hour 0 and 4 in the Saline and RCA-two groups. **c** The mean concentrations of serum iCa from baseline to 4 h in the Saline and RCA-two groups. The change of concentrations of serum-ionized calcium from baseline to endpoint were analyzed with the mixed models for repeated measures with an unstructured residual covariance matrix (main effect of time *P* = 0.193, anticoagulation group *P* < 0.001 and interaction effect *P* = 0.311). **d** The mean concentrations of serum iCa from baseline to 4 h in the RCA-one and RCA-two groups. The change of concentrations of serum-ionized calcium from baseline to endpoint were analyzed with the mixed models for repeated measures (main effect of time *P* = 0.492, anticoagulation group *P* < 0.001 and interaction effect *P* = 0.021). **e** The percentage of hypocalcemia events (iCa^2+^ < 0.9 mmol/L) between the RCA-one and RCA-two groups. **f** The percentage of hypocalcemia events (iCa^2+^ < 0.9 mmol/L) between the Saline and RCA-two groups. (*P* < 0.05, statistical significance). Abbreviations: Saline, saline flushing group; RCA-two, two-stage regional citrate anticoagulation group; RCA-one, one-stage regional citrate anticoagulation group; n.s., no significant statistical differences; iCa, ionized calcium; TSC, trisodium citrate

**Fig. 5:**
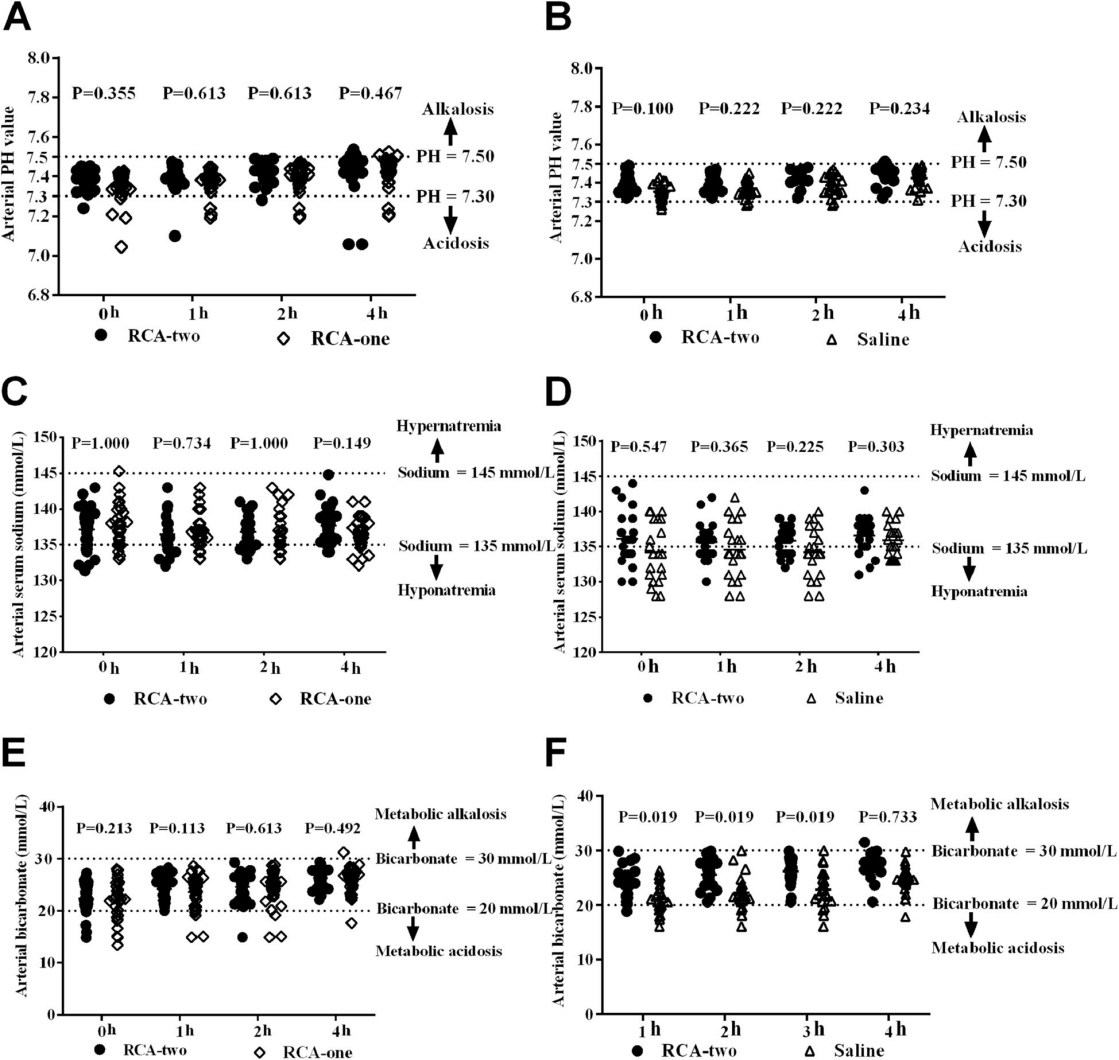
The comparison of values of the internal environmental parameters throughout hemodialysis procedures. PH values between the RCA-two and RCA-one group (**a**) / the Saline group(**b**); Serum sodium values between the RCA-two and RCA-one group (**c**) / the Saline group(**d**); Serum bicarbonate between the RCA-two and RCA-one group (**e**) / the Saline group(**f**). (*P* < 0.05, statistical significance). Abbreviations: Saline, saline flushing group; RCA-two, two-stage regional citrate anticoagulation group; RCA-one, one-stage regional citrate anticoagulation group

The TSC overdose was estimated as the ratio of total calcium to ionized calcium (T/I Ca^2+^) above 2.5. All T/I Ca^2+^ ratios at 0 h were less than 2.5. T/I Ca^2+^ ratios above 2.5 occurred in six of 71 patients (8.45%) at 4 h in the RCA-two group, two of 39 patients (5.13%) at 4 h in the RCA-one group (Fig. 4a) and 0 of 31 patients (0.00%) in the saline group at the end of dialysis (Fig. 4b). There were no significant statistical differences in the occurrence of TSC overdose among groups (*P* > 0.05). The change of ionized calcium serum levels from baseline to 4 h were not significant in two part respectively (main effect of time *P* = 0.193, anticoagulation group *P* < 0.001 and interaction effect *P* = 0.311 in part one, Fig. 4c; main effect of time *P* = 0.492, anticoagulation group *P* < 0.001 and interaction effect *P* = 0.021 in part two, Fig. 4d). Among groups, the serum ionized calcium level was significantly decreased in the RCA-two group than the RCA-one and saline groups at most time points (*P* < 0.05; Fig. 4c and d). Hypocalcemia (iCa^2+^ < 0.9 mmol/L) was only found in a few patients throughout treatment, and there were no statistical differences in the occurrence of hypocalcemia among these groups (P > 0.05; Fig. 4e and f). Moreover, there were no significant internal environment disturbances, including pH values > 7.50, serum bicarbonate > 30 mmol/L, hypernatremia (P > 0.05) in all groups throughout the HD procedures (Fig. 5).

## Discussion

In this multicenter, controlled, prospective, randomized clinical study, we found that our modified regional citrate anticoagulation protocol, namely, RCA-two in a Ca^2+^-containing dialysate, was superior compared to either saline flushing or traditional RCA-one in HD patients with a high bleeding risk. Patients on HD have an increased risk of bleeding caused by uremia-associated platelet dysfunction, other defects of hemostasis, and anticoagulation with heparin [17–19]. Approximately 8–36% of dialysis patients are treated with oral anticoagulants or antiplatelet agents for cardio-cerebrovascular complications [20, 21] with 0.05–0.22 events/year of major bleeding rates worldwide [22, 23]. In addition, perioperative management of HD patients for surgery, e.g., for parathyroidectomy, vascular access surgery, trauma or renal transplantation, has become more and more routine with a systemic anticoagulation contraindication [24]. For the medical insurance policy in China, such a large population may not achieve an adequate dialysis dose after interruption by frequent serious clotting.

Prior clinical studies have explored RCA under different conditions. An open-label single-center prospective clinical trial including 33 patients at risk of bleeding demonstrated that RCA using a Ca^2+^-free dialysate (RCA-Ca0) revealed fewer clotting events than RCA-one (RCA-Ca3), or anticoagulant-free hemodialysis, while Ca^2+^ supplementation is necessary for the RCA-Ca0 group [9]. However, the cumbersome and laborious procedure during RCA-Ca0 had limited its clinical application. Subsequent studies detected an RCA effect in other forms with simpler techniques, for example, a Ca^2+^-containing dialysate or a hemodialysis filtration (HDF) procedure. Evenepoel et al. found that RCA-one for HD was shown to be safe and relatively effective but still exhibited moderate blood clotting compared with heparin (26% vs. 5%, *P* = 0.036) [25]. Later studies found that RCA-one resulted in significant clotting (up to 40%) in the venous bubble trap [26, 27]. However, these observational studies had methodological limitations to some extent. Ponikvar only observed RCA efficacy in HDF, without any contrast or necessary safety assessment. There were no adjustments for gender, age, weight, Ca^2+^, or blood platelets. Generally, RCA-one was inferior in the venous bubble trap, probably caused by an elimination of TSC by the dialyzer and restoration of Ca^2+^ by dialysate.

To solve this problem, we modified the RCA-one technique, that one-quarter of the total TSC was imported from the artery line to the venous bubble trap. The strengths of this study included that it was a multicenter, randomized, prospective clinical trial. For the efficiency analysis, we compared RCA-two with saline flushing and RCA-one, and the anticoagulation superiority was significant in RCA-two group, comparing with the saline and RCA-one group. The anticoagulation effect in the RCA-one group in this study was worse than in previous studies [11, 25]. One possible explanation for the disparity between the results observed in these trials were the methodological differences. Previous studies focused more on dialyzer clotting, but we considered the dialyzer, artery bubble, and venous bubble in this trial because any of the above aspects could interrupt the regular therapy. Another factor might be the different Ca^2+^ concentrations in the dialysate, wherein it was 1.5 mmol/L Ca^2+^ in our study, compared with 1.25 mmol/L Ca^2+^ in previous studies.

Dialysis session length is one of the essential factors associated with all-cause mortality among HD patients [2, 28], and anticoagulation efficiency would theoretically account for the dialysis session length. In this study, results of the ECC survival time indicated that RCA-two would provide the best guarantee of dialysis sufficiency. Frequent ECC high-pressure alarms and circuit replacement would enlarge nurse workloads and economic costs. The efficacy of hemodialysis was also assessed by calculating Kt/V and URR [29]. The Kt/V and URR results in this study demonstrated the best efficacy of the dialysis dose in RCA-two, although the Kt/V and URR were affected by other factors, such as the dialyzer type, blood flow, and access type.

Another strength of this study was that major confounders were analyzed based on the Cox regression models, affirming the independent anticoagulation role of RCA-two (Table 2). The previous trials were weak in this respect. Compared with the RCA-one group, adjusted HR in the RCA-two group was slightly increased from 0.211 to 0.224 in the Cox proportional hazards models. Compared with the saline group, adjusted HR was lower, from 0.222 to 0.184, suggesting that the anticoagulation of RCA-two was more effective after adjusting other factors (Table 2).

Generally, statistical differences in the safety population is not expected given the size sample. So a larger clinical cohort was needed to provide more robust data about the safety population. One-quarter of the TSC was imported back to the systemic circulation in the RCA-two procedure. Is it safe enough, especially in patients with a high risk of bleeding? The TSC is mainly metabolized in the liver and skeletal muscle in HD patients, liberating the Ca^2+^ and rapidly producing bicarbonate [30]. Theoretically, it would not accumulate in patients with normal liver and muscle metabolism. We had excluded patients with a serum bilirubin higher than 60 μmol/L as liver failure. There were six cases in RCA-two (8%) and two cases (5%) in RCA-one with a T/I Ca^2+^ concentration higher than 2.5 at 4 h. We also tested serum citrate concentration using a citrate assay kit, and the average serum citrate concentration of patients with a T/I Ca^2+^ concentration higher than 2.5 was 0.02 mmol/L (Range: 0.01–0.07 mmol/L), within the safety range (< 1.0 mmol/L) according to previous recommendations. The correlation of serum citrate and T/I Ca^2+^ was 0.537 (0.123–0.791), *P* = 0.015 (data not shown). Although several patients were at risk of citrate overdose by the end of therapy, the T/I Ca^2+^ recovery occurred in all of them by the beginning of the next HD session 2 or 3 days later. The exact recovery time is unknown, which is also a limitation of this study.

Because TSC functions by binding Ca^2+^, hypocalcemia is a major complication during RCA, which will subsequently cause hypotension and convulsions and even serious arrhythmia [31]. In the modified RCA-two process, we aimed to simplify the procedure so that Ca^2+^ need not be monitored or supplemented into the systemic circulation. We determined whether the small portion of TSC imported directly back to the body in RCA-two would impact the serum Ca^2+^? As shown in Fig. 4c and d, the average concentration of iCa^2+^ was significantly lower in the RCA-two group, compared with the RCA-one and saline group, but the average concentration was above 0.9 mmol/L throughout the trial. Several patients developed asymptomatic hypocalcemia in RCA-two and RCA-one group demonstrated that patients were endurable with such complication. Hypomagnesemia, hypernatremia, and metabolic alkalosis were rare throughout the trial. Of note is that hyponatremia was observed in several participants, but we theorized that it was partly iatrogenic because we had reduced the concentration of sodium to 137 mmol/L in the dialysate. Other complications (e.g., hypotension, convulsions and hypoglycemia) did not differ among the groups. The major complication was hypotension, and this may be partly related to the larger ultrafiltration per hour with the infusion of TSC.

There are multiple limitations of this study that need to be mentioned. First, a larger clinical cohort is needed to provide more robust data about the safety population mentioned previously. Secondly, due to data limitations, we could not exclude all of the oral and hemostatic anticoagulants that participants were taking as a potential confounder and bias affecting the results [32, 33]. Other additional markers for coagulation activity such as D-dimer and fibrinogen [34, 35], which would be residual confounders, were not tested in this study.

Third, there were several inevitable biases in this trial. All the hospitals participating in this trial were high-grade hospitals in the urban area. The basic homogeneity of the included participants was relatively stable, and the main safety assessment indexes may be better than those in the real world. In this study, participants could voluntarily take part in one to three sessions during the trial, and therefore, for the open label study design, a choice bias exists to some extent; for example, participants with serious clotting may not be willing to continue for another session. To minimize the bias, additional assessment in separated data including only the first session of all participants showed that serious clotting events in the RCA-two group were still significantly lower than in either the RCA-one or saline group. (*P* < 0.05; Additional file 4: Figure S4).

Fourth, to minimize heterogeneity, we did not carry out the research in the real world, limiting its clinical application. For example, we did not observe the effectiveness of the hemodialysis filtration (HDF) or hemofiltration (HF) models, which are also major patterns of blood purification in our country.

## Conclusion

This study demonstrated the superiority of our modified anticoagulation RCA-two over either saline flushing or RCA-one in a population of intermittent HD patients with a high risk of hemorrhage. Our study clarified the effectiveness and safety of RCA-two. RCA-two could provide the best guarantee in dialysis sufficiency with the fewest clotting events and better clinical feasibility. At the same time, rare occurrences of TSC accumulation or other complications were found during the RCA-two procedure. Finally, a larger clinical cohort in the real world should be carried out in the future.

## Supplementary information

**Additional file 1: Figure S1.** Efficacy endpoints in detail. The percentage of sessions with persistent venous pressure (> 200 mmHg), TMP (> 300 mmHg) and visible serious circuit clotting in part one (**A**) and part two (**B**); the clotting scores of ECC in part one (**C**) and part two (**D**). Abbreviations: Saline, saline flushing group; RCA-two, two-stage regional citrate anticoagulation group; RCA-one, one-stage regional citrate anticoagulation group; n.s., no significant statistical differences; TMP, transmembrane pressure.

**Additional file 2: Figure S2.** Correlation coefficients between variables by Spearman’s correlation analysis in part one trial. The main purpose of this analysis was to reduce the possibility that the same associated variable entered the multivariate model simultaneously, the correlation analysis between the factors screened using univariate analysis was performed. If the Spearman’s correlation coefficient between variables exceeds 0.60, only the variables considering being more important on a clinical basis were entered into the multivariate model. Variables with similar functions were chosen using the likelihood ratio test, which favored those variables with higher statistical values. Abbreviations: APTT, Activated partial thromboplastin time.

**Additional file 3: Figure S3.** Correlation coefficients between variables by Spearman’s correlation analysis in part two trial. The main purpose of this analysis was to reduce the possibility that the same associated variable entered the multivariate model simultaneously, the correlation analysis between the factors screened using univariate analysis was performed. If the Spearman’s correlation coefficient between variables exceeds 0.60, only the variables considering being more important on a clinical basis were entered into the multivariate model. Variables with similar functions were chosen using the likelihood ratio test, which favored those variables with higher statistical values. Abbreviations: APTT, Activated partial thromboplastin time.

**Additional file 4: Figure S4.** Efficacy endpoints of the first session of all patients. **(A)** Serious clotting rate in part one and part two trial (9/26(34.62%) vs. 2/26(7.69%), *P* = 0.039; 11/22(50.00%) vs. 1/23(4.35%), *P* < 0.001, respectively). (**B**) The circuit survival time of part one and part two. The median circuit survival time was 240 min (IQR 173 to 240) in the RCA-one group vs 240 min (IQR 240 to 240) in the RCA-two group (*P* = 0.019); The median circuit survival time was 221 min (IQR 170 to 240) in the Saline group vs 240 min (IQR 240 to 240) in the RCA-two group (*P* < 0.001). Abbreviations: Saline, saline flushing group; RCA-two, two-stage regional citrate anticoagulation group; RCA-one, one-stage regional citrate anticoagulation group.

**Additional file 5: Figure S5.** The concentration of iCa^2+^ at several points of the ECC in RCA-tw o (A) and RCA-one (B). **(A)** We innovatively transferred one-quarter of the TSC from the prefilter to the postfilter based on RCA-one, which is called RCA-two in this trial. The concentration of iCa^2+^ at venous bubble trap was reduced significantly. **(B)** We previously tested the concentration of iCa^2+^ at several points of the ECC in RCA-one and found that the concentration of iCa^2+^ is particularly high in the venous bubble trap, which would be the key point of clotting. Abbreviations: RCA, regional citrate anticoagulation; ECC, the extracorporeal circuit; TSC, trisodium citrate.

**Additional file 6: Table S1.** Criteria of clotting scores of extra corporeal circuit at the end of dialysis.

**Additional file 7: Table S2.** Supplemental baseline characteristics.

**Additional file 8: Table S3.** Univariate analysis of influencing factors of serious clotting events.

## Data Availability

The datasets used and/or analyzed during the present study are available from the corresponding author on reasonable request.

## Abbreviations

AEs: Adverse events
APTT: Activated partial thromboplastin time
Ca: Calcium
CI: Confidence interval
CKD: Chronic kidney disease
C_post_: The post-dialysis blood urea (mmol/liter)
C_pre_: The pre-dialysis blood urea (mmol/liter)
CRRT: Continuous renal replacement therapy
ECC: Extracorporeal circuit
HD: Hemodialysis
HDF: Hemodiafiltration
HF: Hemofiltration
HR: Hazard ratio
iCa: Ionized calcium
IQR: Interquartile range
KDIGO: Kidney Disease Improving Global Outcomes
Kt/V: Whole body urea clearance
Mg: Magnesium
n: Number of patients
PES: Polyethersulfone
Q_L_: The lower quartile
Q_U_: The upper quartile
R^2^: Square of correlation coefficient
RCA: Regional citrate anticoagulation
RCA-one: One-stage regional citrate anticoagulation group (Regional citrate anticoagulation has been used in a Ca2+ containing dialysate with prefilter citrate in one arm)
RCA-two: Two-stage regional citrate anticoagulation group (we innovatively transferred one-quarter of the TSC from the prefilter to the postfilter based on RCA-one, which is called RCA-two in this trial)
Saline: Saline flushing group
t: The dialysis session length (hours)
T/I Ca^2+^: Total calcium to ionized calcium
TAT: Triacetate
TMP: Transmembrane pressure
TSC: Trisodium citrate
UF: The total volume of ultrafiltrate (liters)
URR: Urea reduction rate
W: The post-dialysis weight (kg)
