# Supplementary material for: Modified regional citrate anticoagulation is optimal for hemodialysis in patients at high risk of bleeding: a prospective randomized study of three anticoagulation strategies
Source: BMC Nephrol. 2019 Dec 19;20:472. doi: 10.1186/s12882-019-1661-y (PMC6924029; doi:10.1186/s12882-019-1661-y)
Supplement: Supplementary file 6 — Additional file 6: Table S1. Criteria of clotting scores of extra corporeal circuit at the end of dialysis. [file 12882_2019_1661_MOESM6_ESM.doc]

**Table S1 Criteria of clotting scores of extra corporeal circuit at the end of dialysis.**

| **Clotting score** | **Hemofilter** | **Expansion chamber** |
| --- | --- | --- |
| **0** | Clear or No clotting of Hemofilter | Clear or No clotting of expansion chamber |
| **1** | Area of streaky Hemofilter less than 1/3 of total | Volume of thrombus less than 1/3 of expansion chamber |
| **2** | Area of streaky Hemofilter less than 2/3 of total | Volume of thrombus less than 2/3 of expansion chamber |
| **3** | Area of streaky Hemofilter more than 2/3 of total | Volume of thrombus more than 2/3 of expansion chamber |
