# Supplementary material for: Modified regional citrate anticoagulation is optimal for hemodialysis in patients at high risk of bleeding: a prospective randomized study of three anticoagulation strategies
Source: BMC Nephrol. 2019 Dec 19;20:472. doi: 10.1186/s12882-019-1661-y (PMC6924029; doi:10.1186/s12882-019-1661-y)
Supplement: Supplementary file 7 — Additional file 7: Table S2. Supplemental baseline characteristics. [file 12882_2019_1661_MOESM7_ESM.doc]

**Table S2 Supplemental baseline characteristics.**

|  | **Part one** | | |  | **Part two** | | |
| --- | --- | --- | --- | --- | --- | --- | --- |
| **Factors** | **RCA-one(n=39)** | **RCA-two(n=38)** | **P value** |  | **Saline(n=31)** | **RCA-two(n=33)** | **P value** |
| Pre-dialysis AP, median (IQR), mmHg | -80 (-95- -60) | -80 (-99- -60) | 0.723 |  | -90 (-100- -80) | -80 (-90- -70) | 0.057 |
| Pre-dialysis VP, median (IQR), mmHg | 80 (40-90) | 90 (80-100) | 0.151 |  | 90 (80-105) | 80 (60-100) | 0.299 |
| Pre-dialysis TMP, median (IQR), mmHg | 75 (56-80) | 80 (60-81) | 0.128 |  | 80 (70-90) | 70 (60-80) | 0.154 |
| Serum bicarbonate, mmol/L | 21.8 (19.4-23.4) | 22.7 (20.4-24.2) | 0.151 |  | 21.6 (20.5-25.4) | 23.5 (21.6-25.7) | 0.108 |
| P_CO2_, mmHg | 40.3 (35.3-43.4) | 37.8 (34.4-39.2) | 0.041 |  | 39.0 (35.0-40.8) | 40.2 (37.5-42.4) | 0.404 |
| Magnesium, mmol/L | 0.94 (0.85-1.00) | 0.93 (0.86-0.99) | 0.896 |  | 0.94 (0.88-1.01) | 0.86 (0.79-1.01) | 0.364 |
| Glucose, mmol/L | 7.21 (6.06-7.92) | 7.63 (6.85-9.73) | 0.138 |  | 6.37 (5.53-8.10) | 7.10 (6.64-8.28) | 0.827 |
| Potassium, mmol/L | 4.28 (3.67-4.93) | 4.03 (3.44-4.49) | 0.112 |  | 4.81 (4.32-5.59) | 4.31 (3.99-4.86) | 0.089 |
| Serum sodium, mmol/L | 136 (134-138) | 136 (135-137) | 0.810 |  | 134 (131-139) | 136 (134-139) | 0.115 |
| Lactic acid, mmol/L | 1.3 (0.8-1.6) | 1.6 (1.3-2.1) | 0.025 |  | 1.4 (0.9-1.7) | 1.3 (1.2-1.6) | 0.836 |
| Urea nitrogen, mmol/L | 19.6 (16.0-25.6) | 19.7 (14.2-28.2) | 0.518 |  | 22.6 (18.7-30.1) | 22.6 (18.1-25.7) | 0.372 |
| Creatinine, μmol/L | 818.2 (616.8-1122.4) | 840.9 (678.0-991.0) | 0.883 |  | 1064.0 (868.0-1193.0) | 759.0 (676.0-1002.0) | 0.054 |

Abbreviations：VP, venous pressures; TMP, transmembrane pressures; AP, arterial pressures; DBP, diastolic blood pressure; SBP, Systolic blood pressure; AV fistula, arterial- venous fistula; IQR, interquartile range; sd, Standard deviation. Categorical variables were described as frequencies (n) or percentages (%) and analyzed with Pearson’s chi-square or Fisher’s exact test. The Kolmogorov-Smirnov test was used to check the normal distribution of all continuous data. Parametric continuous parameters are expressed as mean±standard deviation and analyzed with unpaired Student’s t-tests; nonparametric continuous parameters are expressed as medians (interquartile range, IQR) and analyzed with the Wilcoxon test.
