# Supplementary material for: Modified regional citrate anticoagulation is optimal for hemodialysis in patients at high risk of bleeding: a prospective randomized study of three anticoagulation strategies
Source: BMC Nephrol. 2019 Dec 19;20:472. doi: 10.1186/s12882-019-1661-y (PMC6924029; doi:10.1186/s12882-019-1661-y)
Supplement: Supplementary file 8 — Additional file 8: Table S3. Univariate analysis of influencing factors of serious clotting events. [file 12882_2019_1661_MOESM8_ESM.doc]

**Table S3 Univariate analysis of influencing factors of serious clotting events**.

|  | **Part one** | | |  | **Part two** | | |
| --- | --- | --- | --- | --- | --- | --- | --- |
| **Factors** | **NON-CLOT (n=62)** | **CLOT (n=15)** | **P value** |  | **NON-CLOT (n=46)** | **CLOT (n=18)** | **P value** |
| **Anticoagulation** |  |  |  |  |  |  |  |
| RCA-two, n (%) | 35 (56.45) | 3 (20.00) | 0.009 |  | 32(69.57) | 1(5.56) | 0.010 |
| RCA-one, n (%) | 27(43.55) | 12(80.00) |  |  |  |  |  |
| Saline, n (%) |  |  |  |  | 14(30.43) | 17(94.44) |  |
| Male sex, n (%) | 31(50.00) | 6(40.00) | 0.487 |  | 23 (50.00) | 11 (61.11) | 0.934 |
| Age, median (IQR),years | 54.00(50.00-64.00) | 57.00(55.00-60.00) | 0.367 |  | 51.50(36.00-68.00) | 60.00(41.00-66.00) | 0.356 |
| Diabetes mellitus, n (%) | 8(12.90) | 3(20.00) | 0.440 |  | 9 (19.57) | 3(16.67) | 0.036 |
| Weight, median (IQR), kg | 54.25 (50.00-61.20) | 59.50 (52.00 - 65.00) | 0.144 |  | 56.95(50.00-60.00) | 62.50(55.00-64.00) | 0.150 |
| Urine, median (IQR), ml | 0.00(0.00-400.00) | 0.00(0.00-200.00) | 0.313 |  | 0.00(0.00-75.00) | 50.00(0.00-300.00) | 0.137 |
| Access type-AV fistula no./catheter no. | 40/22 | 10/5 | 0.876 |  | 32/14 | 10/8 | 0.318 |
| Access site-Left no./right no. | 34/28 | 10/5 | 0.563 |  | 29/17 | 13/5 | 0.495 |
| **Causes of high risk of bleeding** |  |  | 0.776 |  |  |  | 0.129 |
| Preoperative, n (%) | 18(34.62) | 7(28.00) |  |  | 19 (44.19) | 6(28.57) |  |
| Postoperative, n (%) | 15(28.85) | 9(36.00) |  |  | 3(6.98) | 5(23.81) |  |
| Hemorrhage, n (%) | 19(36.54) | 9(36.00) |  |  | 21(48.84) | 10(47.62) |  |
| **Complications** |  |  | 0.845 |  |  |  | 0.752 |
| Dizzy, n (%) | 1(1.61) | 0(0.00) |  |  | 1(2.17) | 0(0.00) |  |
| Convulsions, n (%) | 1(1.61) | 0(0.00) |  |  | 1(2.17) | 1(5.56) |  |
| Hypoglycemia, n (%) | 1(1.61) | 0(0.00) |  |  | 2(4.35) | 1(5.56) |  |
| Hypotension, n (%) | 7(11.29) | 3(20.00) |  |  | 3(6.52) | 0(0.00) |  |
| Chest tightness, n(%) | 3(4.84) | 1(6.67) |  |  | 1(2.17) | 0(0.00) |  |
| Other, n (%) | 1(1.61) | 0(0.00) |  |  | 0(0.00) | 1(5.56) |  |
| Pre-dialysis hemoglobin, median (IQR),g/L | 89.00(83.00-106.00) | 89.00(84.00-98.00) | 0.681 |  | 87.00 (80.00-102.00) | 98.00(94.00- 110.00) | 0.024 |
| Pre-dialysis blood platelets, median (IQR),10^9^/L | 192.00(131.00-252.00) | 210.00(167.00-265.00) | 0.325 |  | 204.00(164.00-224.00) | 187.00(173.00- 226.00) | 0.969 |
| Pre-dialysis WBC, median (IQR), mmol/L | 6.20(5.37-7.10) | 7.45(5.71-8.74) | 0.134 |  | 5.78(4.54-6.61) | 5.78(5.12-7.53) | 0.463 |
| Pre-dialysis prothrombin time, median (IQR), second | 13.75(13.30-14.50) | 13.80(13.30-14.45) | 1.000 |  | 13.40 (12.60-14.30) | 13.65(13.00-14.40) | 0.358 |
| Activated partial thromboplastin time, median (IQR), second | 39.75(37.60-41.70) | 36.90(33.05-39.85) | 0.027 |  | 39.35(36.80-42.60) | 38.95(36.55-42.00) | 0.900 |
| Pre-dialysis median Serum sodium, median (IQR), mmol/L | 137.70(135.60-139.00) | 137.00(135.00-140.40) | 0.966 |  | 135.00(133.00-139.00) | 134.00(131.00-137.00) | 0.338 |
| Pre-dialysis pH value, median (IQR), mmol/L | 7.39(7.35-7.42) | 7.38(7.32-7.41) | 0.271 |  | 7.38(7.35-7.42) | 7.37(7.35-7.39) | 0.175 |
| Pre-dialysis Serum bicarbonate, median (IQR), mmol/L | 22.10(20.40-23.90) | 22.45(18.05-25.70) | 0.631 |  | 23.20(20.70-25.70) | 21.90(20.70-24.50) | 0.145 |
| Pre-dialysis Magnesium, median (IQR), mmol/L | 0.93(0.85-0.99) | 0.94(0.90-1.00) | 0.604 |  | 0.89(0.80-0.99) | 0.97(0.89-1.01) | 0.134 |
| Pre-dialysis Glucose, median (IQR), mmol/L | 7.40 (6.67-9.03) | 7.28 (6.42-9.25) | 0.949 |  | 7.10(6.24-8.28) | 7.96(5.78-8.91) | 0.703 |
| Pre-dialysis Potassium, median (IQR), mmol/L | 4.05(3.49-4.77) | 4.21(3.50-4.81) | 0.119 |  | 4.45(4.16-4.97) | 5.14(4.32-5.66) | 0.289 |
| Pre-dialysis blood ionized calcium, median (IQR), mmol/L | 1.11(1.06-1.17) | 1.10(1.05-1.16) | 0.371 |  | 1.09(1.01-1.19) | 1.14(1.01-1.20) | 0.545 |
| Pre-dialysis total calcium, median (IQR), mmol/L | 2.25(2.05-2.40) | 2.20(2.10-2.40) | 0.596 |  | 2.20(2.10-2.30) | 2.30(2.10-2.50) | 0.459 |
| Pre-dialysis total calcium to ionized calcium (T/I Ca^2+^) | 1.98(1.90-2.11) | 2.07(1.82-2.16) | 0.105 |  | 2.05(1.93-2.14) | 2.02(1.91-2.11) | 0.946 |
| Pre-dialysis Total bilirubin, median (IQR), μmol/L | 8.70(6.90-10.70) | 8.30(6.70-9.20) | 0.241 |  | 8.30(6.00-13.30) | 9.80(6.60-11.20) | 0.817 |
| Pre-dialysis lactic acid, median (IQR), mmol/L | 1.40(1.00-2.00) | 1.40(1.30-1.80) | 0.491 |  | 1.40(1.00-1.60) | 1.50(1.30-1.60) | 0.494 |
| Pre-dialysis PCO_2_, median (IQR), mmHg | 38.60(35.20- 40.90) | 38.45(33.40- 40.70) | 0.541 |  | 40.00(36.90-42.40) | 38.70(35.80-40.20) | 0.297 |
| Pre-dialysis Urea nitrogen, median (IQR), mmol/L | 20.02(15.40- 25.60) | 20.49(15.90- 35.65) | 0.457 |  | 22.60(18.10-25.70) | 25.00(22.50-33.70) | 0.111 |
| Pre-dialysis creatinine, median (IQR), μmol/L | 816.40 (661.10- 1055.00) | 925.00 (703.20- 1100.40) | 0.574 |  | 878.00(658.00-1144.00) | 1064.0(759.00-1113.00) | 0.295 |
| Pre-dialysis HR, median (IQR), bpm | 79.08±11.16 | 81.93±8.81 | 0.383 |  | 80.05±14.39 | 74.86±13.26 | 0.771 |
| Pre-dialysis SBP, median (IQR), mmHg | 150.96±26.25 | 157.93±17.88 | 0.354 |  | 151.37±24.44 | 151.52±22.21 | 0.768 |
| Pre-dialysis DBP, median (IQR), mmHg | 79.96±12.85 | 77.93±8.68 | 0.581 |  | 82.93±15.98 | 78.90±18.29 | 0.336 |
| Pre-dialysis AP, median (IQR), mmHg | -80.00(-98.00- -60.00) | -80.00(-100.00- -40.00) | 0.837 |  | -90.00 (-100.00- -80.00) | -80.00(-100.00- -79.00) | 0.511 |
| Pre-dialysis VP, median (IQR), mmHg | 80.00(70.00-100.00) | 85.00(60.00-100.00) | 0.980 |  | 90.00 (71.00-100.00) | 80.00(63.00-90.00) | 0.185 |
| Pre-dialysis TMP, median (IQR), mmHg | 70.00(60.00-80.00) | 80.00(70.00-83.00) | 0.248 |  | 80.00(60.00-100.00) | 80.00(65.00-90.00) | 0.632 |
| Prescription Ultrafiltration, median (IQR), L | 2.00(1.00-2.80) | 2.00(1.50-2.50) | 0.994 |  | 2.00(1.50-2.50) | 1.75(1.00-2.55) | 0.315 |
| Actual Ultrafiltration, median (IQR), L | 2.00(1.00-2.80) | 1.25(0.80-1.70) | 0.008 |  | 1.80(1.40-2.32) | 1.00(0.62-2.00) | 0.023 |
| Blood flow, median (IQR), mL/min | 200.00 (200.00-230.00) | 200.00(200.00-200.00) | 0.002 |  | 210.00(200.00-220.00) | 200.00(200.00-230.00) | 0.449 |

Abbreviations: IQR, interquartile range; n, patient; Saline, saline flushing group; RCA-two, two-stage regional citrate anticoagulation group; RCA-one, one-stage regional citrate anticoagulation group; TMP, transmembrane pressure; VP, venous pressure; AP, Arterial pressure; HR, Heart rate; bpm, beats per minute; SBP, Systolic blood pressure; DBP, Diastolic blood pressure; PCO2, Carbon dioxide partial pressure; WBC, White Blood Cell. Categorical variables were described as frequencies (n) or percentages (%) and analyzed with Pearson’s chi-square or Fisher’s exact test. The Kolmogorov-Smirnov test was used to check the normal distribution of all continuous data. Parametric continuous parameters are expressed as mean±standard deviation and analyzed with unpaired Student’s t-tests; nonparametric continuous parameters are expressed as medians (interquartile range, IQR) and analyzed with the Wilcoxon test.
